# Alien molluscan species established along the Italian shores: an update, with discussions on some Mediterranean “alien species” categories

**DOI:** 10.3897/zookeys.277.4362

**Published:** 2013-03-15

**Authors:** Fabio Crocetta, Armando Macali, Giulia Furfaro, Samantha Cooke, Guido Villani, Ángel Valdés

**Affiliations:** 1 Stazione Zoologica Anton Dohrn, Villa Comunale, I-80121 Napoli, Italy; 2 Dipartimento di Biologia, Università Roma Tre, Viale Marconi 446, I-00146 Roma, Italy; 3 Department of Biological Sciences, California State Polytechnic University, 3801 West Temple Avenue, Pomona, California 91768-4032, USA; 4 Istituto di Chimica Biomolecolare (CNR), Via Campi Flegrei 34, I-80078 Pozzuoli (Napoli), Italy

**Keywords:** Alien Mollusca, natural entries, translocations, state of knowledge, Italy

## Abstract

The state of knowledge of the alien marine Mollusca in Italy is reviewed and updated. *Littorina saxatilis* (Olivi, 1792), *Polycera hedgpethi* Er. Marcus, 1964 and *Haminoea japonica* Pilsbry, 1895are here considered as established on the basis of published and unpublished data, and recent records of the latter considerably expand its known Mediterranean range to the Tyrrhenian Sea. COI sequences obtained indicate that a comprehensive survey of additional European localities is needed to elucidate the dispersal pathways of *Haminoea japonica*.Recent records and interpretation of several molluscan taxa as alien are discussed both in light of new Mediterranean (published and unpublished) records and of four categories previously excluded from alien species lists. Within this framework, ten taxa are no longer considered as alien species, or their records from Italy are refuted. Furthermore, *Trochocochlea castriotae* Bellini, 1903 is considered a new synonym for *Gibbula albida* (Gmelin, 1791). Data provided here leave unchanged as 35 the number of alien molluscan taxa recorded from Italy as well as the percentage of the most plausible vectors of introduction, but raise to 22 the number of established species along the Italian shores during the 2005–2010 period, and backdate to 1792 the first introduction of an alien molluscan species (*Littorina saxatilis*) to the Italian shores.

## Introduction

Although marine invasions have been well documented all over the world, they are particularly conspicuous in the Mediterranean Sea, mainly due to the high number of vectors of introduction (Galil 2009, 2012; Zenetos et al. 2010, 2012). Moreover, the complex geological history of the region, and the fact that the basin was almost entirely re-colonized naturally by the Atlantic Ocean fauna (Harzhauser et al. 2007) - and this natural interference is still in progress - makes the situation in the Mediterranean even more complex, with the possibility that relatively recent natural dispersals can be interpreted as human-mediated introductions. In this paper we examine some molluscan species from the Mediterranean Sea that appear to be the result of human introductions, with special emphasis on Italy. The previously published state of knowledge of Italian alien molluscan species for the period 2005-2010 (see Crocetta 2011, 2012) is updated based on both molecular data and faunal observations. Additionally, discussions of several taxa belonging to four categories previously excluded by Crocetta (2012) from alien species lists [1- species recorded on the basis of empty shells only; 2- possible cryptogenic or vagrant species (here listed as “Deep-water species, natural dispersers or species with a plausible Atlanto-Mediterranean distribution”); 3- species with a complex/unclear taxonomy; 4- translocations of native Mediterranean species to an area where they previously did not occur], but recently recorded from Italy or included among “Mediterranean aliens” by other authors, are relevant to the entire Mediterranean basin.

## Materials and methods

### Alien species definition and establishment status

The inclusion (or not) of the taxa listed as aliens in the Mediterranean is based on strict accordance to the definition of alien species by the International Union for Conservation of Nature:

‘[non-native, non-indigenous, foreign, exotic] means a species, subspecies, or lower taxon occurring outside of its natural range (past or present) and dispersal potential (i.e., outside the range it occupies naturally or could not occupy without direct or indirect introduction or care by humans) and includes any part, gametes or propagule of such species that might survive and subsequently reproduce.’

Four categories previously excluded (Crocetta 2012), but included by other authors, are discussed. Additionally, an alien species is considered as “established” if, during the surveyed period, at least one self-maintaining population is currently known to occur in the wild (see Crocetta 2012). The definition of cryptogenic species follows Carlton (1996): ‘a species that cannot be included with confidence among native nor among introduced ones.’

### Taxonomy, nomenclature, published and unpublished data collection

A survey of published Mediterranean records of the taxa listed in the present paper was conducted and, where necessary, a full list of synonyms for Mediterranean records is provided. Unpublished records, when available, are listed under each species and come from the examination of preserved specimens, photographs and personal observations obtained from different research projects conducted in several sites along the Italian coasts. Updated taxonomy and nomenclature used follow WoRMS (World Register of Marine Species: last accessed 30 January 2013), unless clearly specified [see *Gibbula albida* (Gmelin, 1791) for the new synonymy proposed].

### DNA extraction, PCR sequencing and data analysis

A total of 8 specimens of *Haminoea japonica* Pilsbry, 1895from two populations in Italy (Lago Fusaro and Lago di Sabaudia)were sequenced for the cytochrome c oxidase I (COI) mitochondrial gene, following methods used in Hanson et al. (2013). Specimens were preserved in 99.8% ethyl alcohol. DNA was extracted from a small foot sample using a hot Chelex® protocol, then amplified by polymerase chain reaction (PCR) using the HCO2198/LCO1490 universal primers for COI (Folmer et al. 1994). PCRs were performed in a 50 μL reaction volume containing 0.25 μL 5U/μL taq polymerase, 5.00 μL 10x buffer, 5.00 μL 25 mM MgCl_2_, 1.00 μL 40 mM dNTPs, 1.00 μL each 10 mM primer, 34.75 μL H_2_O, and 2.00 μL extracted DNA. Reaction conditions involved an initial denaturation of 95°C for 3 min, 35 cycles of 94°C for 45 s, 45°C for 45 s, and 72°C for 2 min, followed by a final elongation step of 72°C for 10 min. PCR products were run on gel electrophoresis to confirm the presence of DNA fragments of appropriate size (700 bp), and positive products were cleaned using Montage PCR Cleanup Kit (Millipore). The DNA concentration of purified samples was then determined using a NanoDrop 1000 spectrophotometer (Thermo Scientific). Sequencing was outsourced to the City of Hope DNA Sequencing Laboratory (Duarte, California, USA) using sequencing buffer BigDye V3.1. Sequences were assembled and edited using the software Geneious pro 4.7.4 (Biomatters Ltd.). Geneious also was used to extract the consensus sequences and to align the sequences using the default parameters. In order to determine the relationships between the haplotypes found in Lago Fusaro and Lago di Sabaudia and other haplotypes found in the non-native range of *Haminoea japonica*, a haplotype network was constructed using TCS 1.21 (Clement et al. 2000) with a fixed connection limit of 100 steps. Sequence data have been submitted to the GenBank databases with accession numbers JX679598-JX679605. Data for non-Italian haplotypes were obtained from Hanson et al. (2013).

## Results and discussion

### Alien molluscan species established along the Italian shores

In order to verify the presence or absence of alien species, a continuous monitoring effort is necessary. Only field surveys may provide such evidence, and the new records of the two species reported below ensure that they fit wel into the category of established species.

**Family LITTORINIDAE Children, 1834**

***Littorina saxatilis* (Olivi, 1792)**

**Unpublished material examined.** *Italy - Veneto*: Malamocco (45°20.35'N, 12°18.80'E), under and on rocks and in crevices at tidal level. Voucher specimens: 05/2009, approx. 50 sps., *legit* F. Crocetta and F. Favero; 06/2009, approx. 100 sps., *legit* F. Crocetta and F. Favero; 03/2010, approx. 20 sps., *legit* F. Crocetta.

**Remarks.** *Littorina saxatilis* (Olivi, 1792) was originally described from the Venice Lagoon (Italy); its disjunct distribution in the eastern and western Atlantic Ocean, Barents Sea, White Sea and Mediterranean (see Panova et al. 2011) led several authors to discuss whether the northern Adriatic population of this species should be considered a recent anthropogenic introduction from northern Europe or a remnant of an earlier wider distribution in the Mediterranean Sea (see Crocetta 2011 and references therein). Only recently, Panova et al. (2011) have shown that the Italian population is a recent introduction, using sequence data from a fragment of the mitochondrial cytochrome b gene. This taxon is now known from the Gulf of Trieste and the Venice area in Italy; a recent record from Monopoli (de Jong 2006) has been questioned (Crocetta et al. 2008). The previously unpublished records listed above confirm the establishment of this alien species along the Italian shores. In addition, *Littorina saxatilis* constitutes the earliest confirmed introduction of an alien species to the Mediterranean Sea; this date was considered to be 1865 (Galil 2012), but is here backdated to at least 1792, confirming alien spreading into the Mediterranean Sea even before completion of the Suez Canal in 1869. Paradoxically, the description date also constitutes the date of its first sighting.

**Family HAMINOEIDAE Pilsbry, 1895**

***Haminoea japonica* Pilsbry, 1895**

Published Mediterranean records

*Haminaea callidegenita* Gibson and Chia, 1989 (sic!) - Alvarez et al. 1993: 59–65 (figures 1–10).

*Haminoea callidegenita* (Gibson & Chia, 1989) - Delongueville and Scaillet 2007: 52–53 (figure 8).

*Haminoea* cf.* japonica -* Cossignani and Ardovini 2011: 375 (figures a, b).

**Unpublished material examined.** *Italy - Latium*: Lago diSabaudia (S2: 41°17.58'N, 13°1.06'E; S3: 41°17.58'N, 13°1.21'E; S4: 41°16.13'N, 13°2.23'E; S5: 41°15'N, 13°2.33'E), low depth on mud and amidst algae. Very common in the area during observations and samplings held from April 2007 to May 2012 (A. Macali, pers. obs) - Voucher specimens: 29/05/2007, approx. 30 sps. and egg masses, *legit* A. Macali; 05/02/2008, around 50 sps. and egg masses, *legit* A. Macali; 06/05/2008, approx. 50 sps. and egg masses, *legit* A. Macali; 24/11/2009, approx. 50 sps. and egg masses, *legit* A. Macali; 24/05/2010, approx. 100 sps. and egg masses, *legit* A. Macali; 21/04/2012, approx. 100 sps. and egg masses, *legit* A. Macali and C. Smriglio (4 sps. used for molecular analysis - JX679602-JX679605).

**Figure 1.**
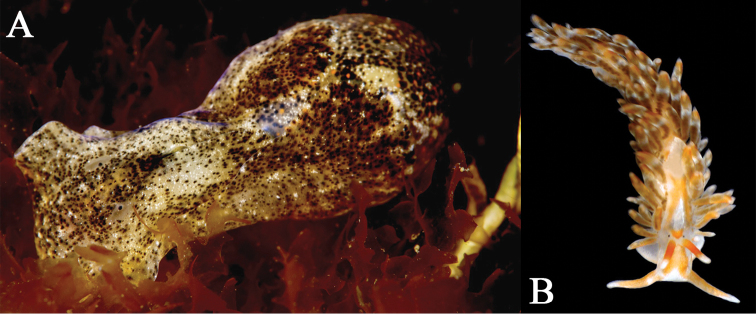
A. *Haminoea japonica* Pilsbry, 1895 from Lago Fusaro, 17/02/2012, approx. 15 mm. Photo: Guido Villani. B. *Anteaeolidiella foulisi* (Angas, 1864) from Lago di Sabaudia, April 2009, 13 mm. Photo: Paolo Mariottini.

*Italy - Campania*: Casina Vanvitelliana, Lago Fusaro (FuI: 40°49.16'N, 14°3.53'E), low depth on mud and amidst algae. Voucher specimens: 27/01/2012, approx. 100 sps. and egg masses, *legit* F. Crocetta; 17/02/2012, 12 sps. and egg masses, *legit* F. Crocetta and G. Villani (Fig. 1A); 25/02/2012, approx. 200 sps. and egg masses, *legit* F. Crocetta (4 sps. used for molecular analysis - JX679598-JX679601); 29/03/2012, approx. 50 sps. and egg masses, *legit* F. Toscano.

**Remarks.** According to Hanson et al. (2013) *Haminoea japonica* Pilsbry, 1895 is native to Japan and Korea. This species has recently spread to the Pacific coast of North America (Gosliner and Behrens 2006; Hanson et al. 2013) as well as into Europe (Atlantic coasts of France and Spain and the Venice-Ravenna area in the northern Adriatic Sea), presumably with imports of bivalves for commercial aquaculture (Cervera et al. 2004; Zenetos et al. 2010; Occhipinti-Ambrogi et al. 2011; Crocetta 2012; Hanson et al. 2013), whilst the record previously reported by Cervera et al. (2004) and Templado et al. (2011) from the Straits of Gibraltar is based on a bibliographic misreading of Álvarez Orive (1994) and is not included here. To date, non-native populations of *Haminoea japonica* are known from areas with relatively cold winter water temperatures, and Hanson et al. (2013) suggested the possibility that the potential spread of *Haminoea japonica* to other regions with exotic bivalve aquaculture facilities (such as southern California and the Hawaiian Islands) might be hampered by warm winter temperatures. That would confirm the temporary nature of the populations of this species in the Mediterranean Sea, where it has been considered as not established, the Venice-Ravenna area populations being considered extinct in 2001–2002 (Crocetta 2012).

**Figure 2.**
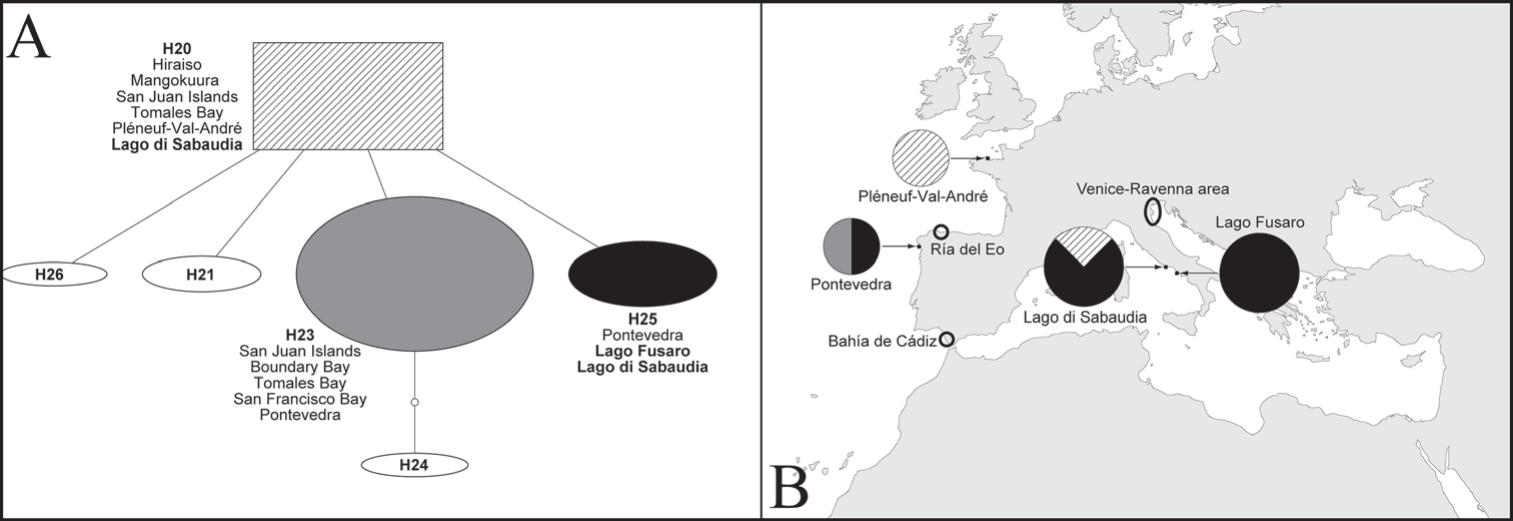
A. Haplotype network of non-native haplotypes of *Haminoea japonica* Pilsbry, 1895 (haplotype IDs from Hanson et al. 2013). Rectangular haplotype (H20) is most ancestral. Sizes of the haplotype icons are proportional to the total number of individuals sequenced (data from Hanson et al. 2013 and present paper). B. Known European records of *Haminoea japonica*, including new collection localities. Sizes of the pie charts are proportional to the number of individuals sequenced from the locality. Pie charts and patterns within indicate the proportion of different haplotypes found in each locality.

However, the recently discovered populations from Lago di Sabaudia (Latium, Tyrrhenian Sea) since 2007 and from Lago Fusaro during 2012 (Figure 1A) considerably expand its known Mediterranean distribution to the Tyrrhenian Sea, and suggest that non-native genotypes can become established in areas warmer than previously thought. Sequence data obtained from Italian specimens are similar to those obtained for other specimens found in the non-native range of *Haminoea japonica*: two distinct haplotypes for COI were detected in Italy, H20 and H25 (according to the haplotype nomenclature by Hanson et al. 2013). H20 was found only in Lago di Sabaudia, whereas H25 in both Lago di Sabaudia and Lago Fusaro (Figure 2). Hanson et al. (2013) also found H20 in north-eastern Japan (the source of the non-native populations), the Pacific coast of North America and France, whereas H25 was previously known only from Spain. This suggests the possibility that *Haminoea japonica* is still spreading and could potentially colonize other areas in the Mediterranean, but it can also suggest that the species is already widespread in the region but remains undetected due to scarcity of faunal studies and experts in opisthobranch taxonomy in areas where mariculture is practiced.

**Discussions on previously excluded categories**

**Species recorded on the basis of empty shells only**

The durable composition of molluscan shells, primarily made of calcium carbonate, often allow the study of local assemblages without sacrificing living animals, offering reliable data on taxa distributions and the ability to study populations qualitatively. Data obtained from the study of empty shells, however, should always be complemented by a very critical approach, and the three taxa cited below are a good example of this. They have been recorded on the basis of worn empty shells only, found in bioclastic sediments trawled at depths of 400-500 m off Latium in 2007 (Perna 2012; E. Perna, pers. comm.). The presence of three Lessepsian shallow water species in the central Tyrrhenian Sea, at such depths, is indeed puzzling. However, because contamination of the samples cannot be excluded, we hereby prefer to exert caution and exclude them from the alien species list until further findings, including that of living specimens, confirm their presence in Italy.

**Family SCALIOLIDAE Jousseaume, 1912**

***Finella pupoides* A. Adams, 1860**

**Remarks.** *Finella pupoides* Adams, 1860 is an Indo-Pacific taxon well established along the eastern Mediterranean shores up to Turkey (Zenetos et al. 2004). A recent record from Italy, based on a shell sampled in 1999 in the Gulf of Taranto, has never been followed by further records either of shells or living specimens (Trono 2006; Crocetta 2012; D. Trono, pers. comm.). It has been recently recorded from Latium, also on the basis of eight shells (Perna 2012; E. Perna, pers. comm.).

**Family CERITHIIDAE Fleming, 1822**

***Clathrofenella ferruginea* (A. Adams, 1860)** *sensu* Perna (2012)

**Remarks. **Four empty shells of a taxon belonging to Cerithiidae have been recently recorded for the first time from Italy (off Latium) as *Clathrofenella ferruginea* (A. Adams, 1860) (Perna 2012; E. Perna, pers. comm.). However, these worn specimens may belong to *Cerithidium diplax* (Watson, 1886) (J.J. Van Aartsen, pers. comm.), one of the two Mediterranean Lessepsian immigrants previously misidentified as *Cerithidium ferruginea* (see Van Aartsen 2006), but due to the state of the specimens a positive identification is unlikely.

**Family VENERIDAE Rafinesque, 1815**

***Timoclea roemeriana* (Issel, 1869)** *sensu* Perna (2012)

**Remarks.** Two loose valves of a bivalve belonging the genus *Timoclea* have been recently recorded for the first time from Italy (off Latium) as *Timoclea roemeriana* (Issel, 1869) (Perna 2012; E. Perna, pers. comm.). This is a Lessepsian species recently reported both as *Timoclea marica* (Mienis 2004, Cecalupo et al. 2008, Zenetos et al. 2010) and *Timoclea roemeriana* (Huber 2010), known from the Mediterranean basin only from the deep eastern shores and from Tunisia (see Cecalupo et al. 2008).

### Deep-water species, natural dispersers or species with a plausible Atlanto-Mediterranean distribution

After the complete re-establishment of the Atlanto-Mediterranean connection, dating approximately 5.33 million years ago, no evidence of further closing of the Strait of Gibraltar exists (Loget and Van Den Driessche 2006), and most of the Mediterranean fauna and flora migrated from the Atlantic Ocean throughout the Gibraltar Strait by natural dispersal. Species widespread in the Atlantic Ocean, or originally described from that area but subsequently recorded from the Mediterranean Sea, have often been interpreted as alien species by some authors (see discussions in Galil 2009, 2012). Unless human-induced activity is clearly involved, many of these records are most likely the result of natural dispersal and their inclusion among aliens can only be justified relative to time, with older records labelled as “native.” However, there are major limitations with this approach: i) it is problematic to determine what constitutes an old or a new introduction, as well as developing objective criteria to establish a temporal boundary between them; ii) it forces researchers to consider biological processes as static, as opposed to the ongoing complex dynamics of populations and ecosystems. Additional biases may be caused by difficulties in the determination of a correct introduction date. Even if it may be relatively straightforward for easy-to-identify, conspicuous, shallow water species, it can be very challenging for pelagic, bathyal or taxonomically difficult species. The five taxa discussed below were recently considered alien species, both deliberately and by mistake.

**Family CIMIDAE Warén, 1993**

***Cima apicisbelli* Rolán, 2003**

**Remarks. ***Cima apicisbelli* Rolán, 2003 has been recently described from Dakar (Senegal, Atlantic Ocean) on the basis of differences in shell and protoconch sculpture with the closely related *Cima cylindrica* (Jeffreys, 1856) (see Rolán 2003). Its presence in the Mediterranean Sea has been first reported from the Gulf of Valencia (Oliver Baldoví 2007, Cossignani and Ardovini 2011) and then from Acitrezza (eastern Sicily, Italy), where Scuderi and Criscione (2011) interpreted it as an alien species, presumably introduced by the discard of marine market animals. However, Gofas (2011), reporting additional specimens from Andalusia where the species is distributed throughout the region, provided additional data to clarify this issue, or rather to cast doubt on the inclusion of *Cima apicisbelli* in the list of alien species. It is, in fact, impossible to determine whether: i) it is native to the Mediterranean Sea and naturally spread into the Atlantic Ocean; ii) it is native of the Atlantic Ocean, but might have an older Atlanto-Mediterranean distribution, overlooked in the Mediterranean Sea until recently due to small size and similarities with other local similar species; or iii) is native of the Atlantic Ocean, but has been introduced into the Mediterranean by human activities. With the available data, *Cima apicisbelli* better fits the definition of a cryptogenic species rather than an alien one.

**Family SEPIOLIDAE Leach, 1817**

***Stoloteuthis leucoptera* (A.E. Verrill, 1878)**

and

**Family CYCLOTEUTHIDAE Naef, 1923**

***Cycloteuthis sirventi* Joubin, 1919**

**Remarks. **The deep water sepiolid *Stoloteuthis leucoptera* (A.E. Verrill, 1878), described from the Gulf of Maine and then recorded from the eastern Atlantic Ocean and the Mediterranean Sea (Degner 1925; Orsi Relini and Massi 1991; Villanueva and Sánchez 1993), as well as the teuthoid squid *Cycloteuthis sirventi* Joubin, 1919, a taxon described from off Madeira (Joubin 1919), have been recently listed as Mediterranean aliens by Bello (2011), following the definition of alien species used by Relini (2008) (natural range expansions + human mediated introductions). As a “natural range expansion” in the Mediterranean Sea is more likely than an introduction throughout a human-induced activity, also suggested by Bello (2011), who considered Mediterranean records as “natural range expansion” and “stray specimens,” they are here excluded from alien species.

**Species with a complex/unclear taxonomy**

**Family AEOLIDIIDAE Gray, 1827**

***Anteaeolidiella foulisi* (Angas, 1864)**

Published Mediterranean records

*Aeolidiella takanosimensis* Baba, 1949 (sic!) - Schmekel 1968: 122, 145.

**Figure 3.**
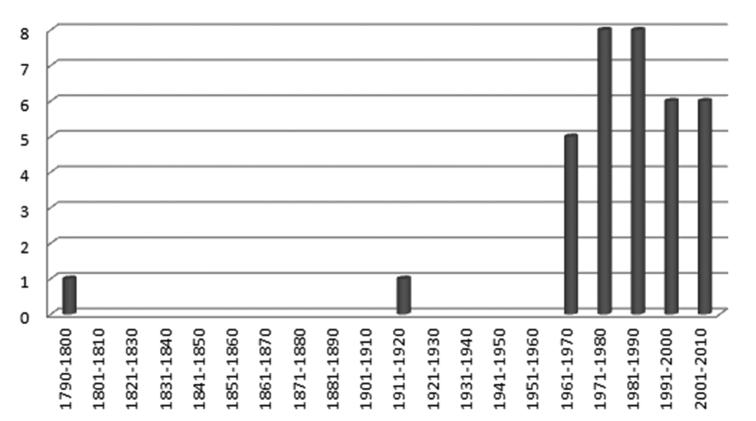
The rate of reporting (as number of species per decade) of alien molluscan species from the Italian territorial seawaters.

*Aeolidiella orientalis takanosimensis* Bergh, 1888 (sic!) - Schmekel and Portmann 1982: 226-228, 352-352 (figures 3–4), 376-377 (figure 10).

*Aeolidiella indica* Bergh, 1888 - Sammut and Perrone 1998: 232, 237; García-Gómez 2002: 258 (figure 130).

**Unpublished material examined.** *Italy - Latium*: Canale Romano, Lago di Sabaudia (S6: 41°15.03'N, 13°2.35'E; S7: 41°14.9'N, 13°2.35'E), 2.5 m depth under stones. Very common in the area during samplings conducted from April 2009 to September 2011 (Fig. 1B) (A. Macali, pers. obs). Voucher specimens: April 2011: 3 sps. and egg masses, *legit* A. Macali; 16/11/2011: 5 sps. and egg masses, *legit* A. Macali.

*Italy - Campania*: Canale Est di Maremorto di Miseno (40°47.55'N, 14°4.68'E), low depth under stones, 30/09/2011: 5 sps. and egg masses, *legit* G. Villani; 04/10/2011: 29 sps. and egg masses, *legit* G. Villani; 01/01/2012: 6 sps., *legit* G. Villani.

**Remarks. ***Anteaeolidiella foulisi* (Angas, 1864) -senior synonym of *Anteaeolidiella indica* (Bergh, 1888), following Burn (2006) - is found throughout the tropical Indo-West Pacific and as far south as northern New Zealand. However, it also occurs in the Atlantic Ocean (e.g. see Marcus and Marcus 1967) and in the Mediterranean Sea, where it is known on the basis of three records (a total of approx. 10 specimens), supposedly introduced by shipping transport (Zenetos et al. 2004, García-Gómez et al. 2011, Crocetta 2012) or via an unknown vector (Sciberras and Schembri 2007, Occhipinti-Ambrogi et al. 2011).

Records from Maremorto di Miseno (Campania, Tyrrhenian Sea) confirm the presence of this species at the same sampling site after approximately 40 years after the last records from Italy, while those from Lago di Sabaudia (Latium, Tyrrhenian Sea) constitute the first record from the area and the only other known Italian site where the species has been reported. Unpublished Italian records reported here, as well as recent records from the Atlantic Ocean (Domínguez et al. 2008, Padula et al. 2011), cast doubts on its inclusion in the list of alien species. According to Willan and Coleman (1984), the widespread distribution of this species is likely due to shipping introductions, but the intraspecific external colour pattern variation recently noted within its wide distributional range, as well as extreme differences between egg masses reported from different localities (Nudi-Pixel web-based identification tool: last accessed 30 January 2013) suggest that *Anteaeolidiella foulisi* could constitute a complex of several distinct species. Additionally, the striking similarities between Atlantic and Mediterranean animals versus other worldwide populations suggest that Mediterranean specimens may simply belong to a species with a conceivably Atlanto-Mediterranean range.

**Family CRANCHIIDAE Prosch, 1849**

***Megalocranchia* sp.**

**Remarks.** The genus *Megalocranchia* includes several species widespread all over the world (Voss 1980). The taxonlisted here as *Megalocranchia* sp. has been recorded in the Mediterranean Sea only on the basis of a photograph of a single adult specimen, which unfortunately was not preserved, and, therefore, cannot be positively identified (Bello 2011). Despite this, it has been recently listed as a Mediterranean alien by Bello (2011). The possibility that this record actually represents an undescribed Mediterranean species is high (as well as a natural range expansion within the Mediterranean Sea of a yet-to-be-described species), and its inclusion among alien species (whatever definition is used) is entirely speculative.

**Translocations of native Mediterranean species to an area where they previously did not occur**

Mediterranean translocations (species introduced from elsewhere within the Mediterranean) have been recently listed as alien species when the introduction event was unmistakable (Zenetos et al. 2010, 2012). With regard to Mediterranean molluscan species, three species were first considered as such by Zenetos et al. (2010): *Siphonaria pectinata* (Linnaeus, 1758), *Gibbula albida* (Gmelin, 1791) and *Echinolittorina punctata* (Gmelin, 1791). These were reduced to two species with the exclusion of *Echinolittorina punctata* by Zenetos et al. (2012); among these, only *Gibbula albida* is known from Italy.

**Family TROCHIDAE Rafinesque, 1815**

***Gibbula albida* (Gmelin, 1791)**

(** =** *Trochocochlea castriotae* Bellini, 1903, new synonym)

**Remarks. ***Gibbula albida* (Gmelin, 1791) has been considered a native species to the Adriatic Sea, but an alien in the western Mediterranean Sea due to recent introductions into the Ebro Delta (Spain) and the French Mediterranean lagoons (see Zenetos et al. 2010). In Italy, it has been considered a translocated species through aquaculture into Laguna di Caprolace (Italy, Latium, Tyrrhenian Sea) (see Bini 1983), thus suggesting that the western Mediterranean shores may have really been only recently colonized by this species.

This taxon, however, was originally described with no type locality (Gmelin 1791), and has been known for centuries to occur commonly in the Adriatic Sea (e.g. Cantraine 1835 as *Trochus bornii* sp. n., Nardo 1847 as *Trochus clodianus* sp. n.), as suggested by Zenetos et al. (2010). It currently ranges from the Black Sea to the North Atlantic Ocean (whether native or introduced), including several old and recent confirmed sightings from the eastern Mediterranean, as well as fossil records from the area (e.g. Deshayes 1833 as *Trochus magulus* sp. n., Rolán et al. 1985, Tenekides 1989, Bachelet et al. 1990, Delamotte and Vardala-Theodorou 1994, Delemarre and Le Neuthiec 1995, Grossu 1999, Morhange et al. 2000, Demir 2003, Anistratenko 2005, Ateş et al. 2007, Stiner 2010). In addition to early records from the Black Sea area and the deep eastern Mediterranean shores, the presence of this species in the Italian Ionian Sea and several western Mediterranean sites also dates back centuries (e.g.: Leghorn: Philippi 1836 as *Trochus biasoletti* sp. n.; Aci Trezza: Aradas and Benoit 1876; Antibes and Nice: Granger 1884 as *Trochus magulus*). Additionally, the taxon *Trochocochlea castriotae* Bellini, 1903 was described by Bellini (1903) on the basis of abundant live material collected from Maremorto di Miseno (Italy, Campania, Tyrrhenian Sea). Although no type material of this species is known to occur in Museo Zoologico, Centro Museale Università degli Studi di Napoli Federico II (Naples, Italy) (N. Maio, pers. comm.), nor in the Museum of the Stazione Zoologica Anton Dohrn di Napoli (Naples, Italy) (A. Travaglini, pers. comm.), and every attempt to trace it was unsuccessful (F. Crocetta, unpublished data), “the canaliculate suture and turriculated whorls,” as well as the image included in the original description (page 23, figs 3a and 3b), allow us to determine with confidence that *Trochocochlea castriotae* Bellini, 1903 is a junior synonym of *Gibbula albida* (Gmelin, 1791).

Although a translocation into the western Mediterranean from the Adriatic is possible, but may have happened earlier than the recent records from France and Spain cited by Zenetos et al. (2010), and taking into account previous reports, we hereby prefer to exclude it from the Italian alien species list. Molecular data is necessary to elucidate whether past and current western Mediterranean distributions of *Gibbula albida* are due to human activities.

## Conclusions

The last decades have seen an ever-increasing worldwide scientific emphasis on biological pollution. Marine alien species feature among the qualitative descriptors of good environmental status in the EU’s Marine Strategy Framework Directive. In this view, alien species inventories play important roles in informing regional policy and management decisions, as well as in identifying resource priorities. The scientific community is called upon to pay particular attention to their accuracy and veracity (Galil 2012). A first attempt to monitor alien molluscan species recorded along the Italian shores, based on the examination of fresh material combined with a critical bibliographic review, was only recently conducted (Crocetta 2012: data obtained until 2010), stating the number of confirmed recorded live alien species to be 35 taxa, and the number of established ones 19 taxa. Data presented here leave unchanged the number of alien molluscan taxa recorded from Italy as 35 - one species, *Littorina saxatilis* (Olivi, 1792) is added but one, *Anteaeolidiella foulisi* (Angas, 1864) is removed - as well as the percentage of the most plausible vectors of introduction, but raise to 22 the number of established species along the Italian shores during the 2005–2010 period (60% of the confirmed recorded ones), and backdates to at least 1792 the first introduction of an alien molluscan species to the Italian shores (see Table 1 and Figure 3). The establishment and maintenance of a comprehensive network of observatories across the Mediterranean is the only viable mechanism with which to monitor changes in species composition taking place all over the basin. In particular, molluscs are currently known to make the largest contribution to the number of documented alien species in the Mediterranean (31% of approx. 660 species: Galil 2012), but this number is expected to increase due to the constant monitoring by researchers, amateur malacologists and diver-photographers, who can potentially detect the presence of new alien species misidentified as native ones in their databases, samples or private collections. Finally, our knowledge on Mediterranean bioinvasions is likely to increase with the use of molecular tools to elucidate population structures of cryptogenic species and to confirm identifications of taxonomically difficult species.

**Table 1.** Alien Mollusca established from the territorial seawaters of Italy during the 2005-2010 period - data after Crocetta (2011, 2012), Keppel et al. (2012) and present paper.

**Taxa**
*Cerithium scabridum* Philippi, 1848
*Littorina saxatilis* (Olivi, 1792)
*Rapana venosa* (Valenciennes, 1846)
*Haminoea cyanomarginata* Heller & Thompson, 1983
*Haminoea japonica* Pilsbry, 1895
*Aplysia dactylomela* Rang, 1828
*Syphonota geographica* (Adams & Reeve, 1850)
*Bursatella leachii* Blainville, 1817
*Polycera hedgpethi* Er. Marcus, 1964
*Melibe viridis* (Kelaart, 1858)
*Godiva quadricolor* (Barnard, 1927)
*Anadara transversa* (Say, 1822)
*Anadara kagoshimensis* (Tokunaga, 1906)
*Brachidontes pharaonis* (P. Fischer, 1870)
*Arcuatula senhousia* (Benson in Cantor, 1842)
*Limnoperna securis* (Lamarck, 1819)
*Pinctada imbricata radiata* (Leach, 1814)
*Crassostrea gigas* (Thunberg, 1793)
*Fulvia fragilis* (Forsskål, 1775)
*Theora lubrica* Gould, 1861
*Venerupis philippinarum* (Adams & Reeve, 1850)
*Mya arenaria* Linnaeus, 1758
